# Influence of Substrate Inclination Angle on Deposition Morphology and Interfacial Microstructure During TIG-Based Wire Arc Additive Manufacturing of Steel/Tin Bimetallic Structures

**DOI:** 10.3390/ma19173617

**Published:** 2026-08-25

**Authors:** Yubin Zhang, Huomei Zhu, Xiaoyun Zhao, Zhiqiang Li, Jun Du

**Affiliations:** 1 College of Mechanical Engineering, Ningbo University of Finance & Economics, Ningbo 315175, China; zhuhuomei@nbufe.edu.cn (H.Z.); zhaoxiaoyun@nbufe.edu.cn (X.Z.); 2 School of Materials and New Energy, Ningxia University, Yinchuan 750021, China; 15109580636@sohu.com

**Keywords:** TIG-based additive manufacturing, bimetallic structure, interfacial microstructure, deposition morphology

## Abstract

**Highlights:**

- Steel/tin bimetallic structures were fabricated using TIG-based wire arc additive manufacturing.
- A non-uniformly distributed intermetallic compound layer at the steel/tin interface was observed.
- The main phases at the steel/tin interfaces consist of matrix phases and intermetallic compounds.
- Increasing the substrate inclination angle induces prominent grain refinement at the bimetallic interface.

**Abstract:**

Steel/tin bimetallic components fabricated using traditional casting processes have inherent drawbacks, including complicated preparation procedures and a relatively low interfacial bonding strength. To efficiently create metallurgical composite steel/tin bimetallic structures under complex service conditions, we utilized TIG-based additive manufacturing with front wire feeding to prepare the components. The effects of the substrate inclination angle on the macroscopic morphology of the deposited layer, interfacial phase composition, the growth behavior of intermetallic compounds (IMCs) at the bimetallic interfaces, and interfacial mechanical properties were investigated. Our results show that macro-structural defects like cracks, voids and pores were not observed at the steel/tin interfaces. The grains of the interface IMCs were mainly composed of Fe_3_Sn, FeSn_2_ and FeSb_2_ phases; Fe-rich microspheres were dispersed inside the deposited tin layer. Under horizontal substrate conditions, deposited layer morphology and IMC layer thickness presented symmetric distributions. When the inclination angle of the substrate reached 30°, the deposited layers exhibited an asymmetric teardrop morphology, resulting in an increased layer height and width and penetration depth. Meanwhile, tin alloy grains were significantly refined; more high-angle grain boundaries (HAGBs) were formed at the spreading fronts of molten droplets. Tin alloy hardness was improved via synergistic dispersion and grain boundary strengthening. This work reveals the inclination–morphology–microstructure–property correlation, fills the research gap in inclined substrate arc additive manufacturing of steel/tin bimetals, and provides a theoretical foundation for engineering applications.

## 1. Introduction

In the field of heavy machinery manufacturing, with the iterative upgrading of equipment technologies, industries such as automotive, shipbuilding and wind power generation have imposed increasingly stringent requirements on the load-bearing strength and wear resistance of sliding bearings [1,2]. Due to their varied performance advantages, dissimilar material composite structures have become the preferred approach to overcoming the performance bottlenecks of single materials and satisfy the demands of high-end transmission components [3,4]. Steel/tin bimetallic structures utilize C45 steel as a matrix in order to bear high loads, while the surface composite layer of tin-based Babbitt alloy provides excellent lubrication and anti-friction properties, making these materials particularly suitable for use in key components such as sliding bearings and gears [5,6]. The quality of interfacial metallurgical bonding directly determines the service life and reliability of components, making it a prerequisite for ensuring the stable operation of equipment. At present, the traditional solid–liquid composite casting process is widely used in industry to fabricate steel/tin bimetallic structures [7]. Although capable of large-area composite fabrication, this process is plagued by disadvantages including complicated procedures and long pretreatment cycles, which can easily lead to interfacial gas gaps, inclusions and thick intermetallic compound (IMC) layers. When this occurs, interfacial bonding strength becomes insufficient for meeting the performance criteria of high-end equipment [8]. In view of these disadvantages, it is vital to develop a new direct metallurgical composite forming process for steel and tin. Optimizing process parameters to precisely control the interfacial bonding state enables the efficient fabrication and application of high-performance steel/tin bimetallic structures, thereby comprehensively improving component service life and reducing their operation and maintenance costs.

As an epoch-making innovative manufacturing technology, multi-material additive manufacturing relies on its unique deposition process to precisely regulate the microstructural and mechanical properties of materials, and is capable of forming components with complex shapes. Accordingly, it has become a research hotspot in the field of advanced forming manufacturing [9,10]. Based on variations in their distribution morphology, the bonding modes of multi-material metal composites can be classified into direct, gradient and interlayer bonding [11,12]. Three AM processes have been proven to be effective in manufacturing single metallic materials and are currently being rapidly developed for use in multi-material metal additive manufacturing (MMAM): (i) laser-directed energy deposition (LDED), (ii) laser-based powder bed fusion (LPBF) [13,14] and electron beam powder bed fusion (EB-PBF) [15,16], and (iii) wire arc AM (WAAM) [17,18,19]. Of these, WAAW and LDED achieve higher deposition rates, exhibit minimal cross-contamination between multiple materials, and are suitable for large-scale component manufacturing. Moreover, WAAM can operate in atmospheric environments and is flexible in terms of the equipment used, making it suitable for forming special-shaped components [20]. This technology thus provides a more feasible route for the efficient and high-quality fabrication of steel/tin bimetallic structures.

Despite the great progress made in MMAM research, critical challenges include heterogeneous thermal properties (coefficient of thermal expansion [CTE], melting temperature, thermal conductivity, and laser absorptivity) and complex behavior at the interfaces of dissimilar materials [9]. In recent years, considerable progress has been made in WAAM deposition technology for tin alloy forming. Chen et al. [21] proposed a new method to eliminate liquid metal embrittlement cracks during tin bronze arc cladding on steel sheets; they found that cracks were generated in bare steel sheets, but not in those plated with nickel. Zhao et al. [22] fabricated tin alloys with varying zinc contents on steel substrates via GMAW and revealed that increasing Zn content contributed to grain refinement and a more uniform distribution of SnSb and Cu_6_Sn_5_ phases, accompanied by a significant improvement in the hardness and tensile strength of the deposited layers. Similarly, He et al. [23] deposited tin alloys on the surface of AZ91D magnesium alloys via TIG and explored the effects of deposition current on the microstructure and tribological properties of the deposited layers. The results demonstrated that a rise in deposition current led to a remarkable increase in the friction coefficient and wear loss, along with deteriorated wear resistance; the primary wear mechanisms were abrasive wear and adhesive wear.

The abovementioned studies have laid a critical foundation for tin alloy WAAM, with most investigations performed under horizontal substrate conditions. However, in practical scenarios, numerous complex structures deposited for AM, such as cantilevers, circles and lateral extensions, necessitate material deposition on non-horizontal surfaces. Inclined substrate surfaces tend to alter the distribution of the heat flux source and destabilize the molten pool, thereby triggering morphological distortion of the deposited layers and deterioration of the forming quality.

At present, certain foundations have been established for research into the working conditions for inclined substrates in laser additive manufacturing. For instance, He et al. [24] investigated the influence of the substrate inclination angle on forming morphology during laser melting deposition (LMD) additive manufacturing; they found that the height and displacement offset of the deposited layers increased with laser power and powder feeding rate. A larger substrate inclination angle led to a slight decrease in deposition height and an increase in displacement offset, whereas the substrate inclination angle showed negligible effects on the tensile and bending strengths. Meanwhile, Zhou et al. [25] successfully deposited 316L stainless steel on inclined substrates via laser-directed energy deposition (LDED), and found that the width of the deposited layers increased with the substrate inclination angle, while the layer height showed no obvious variation and the sidewall roughness rose significantly. Li et al. [26] demonstrated microstructural characteristics and mechanical property evolution during titanium alloy laser welding under inclined substrate conditions. Compared with laser butt welding on horizontal substrates, both the tensile strength and elongation of the welded joints decreased under inclined substrate conditions. In terms of arc discharge mechanisms, Saifutdinov [27] developed a self-consistent unified model of direct current (DC) gas discharges to describe the processes that occur in both the discharge gap and in the electrodes, finding that in the cathode layer, Joule heating through the ion current plays a dominant role in gas heating. Benilov et al. [28] adopted an extended model of non-equilibrium near-cathode high-pressure plasma layers to successfully simulate the ignition of a 200A arc with a tungsten cathode and a copper anode in atmospheric-pressure argon. Three arc ignition phases were identified, and reasonably accurate descriptions of the glow-to-arc transition and subsequent arc development were provided by the extended model. Baeva and Uhrlandt [29] reviewed the applications and characterization of micro-sized direct current arcs, presenting an overview of microarc models comprising a variety of gases, both refractory and nonrefractory cathode operation, and the complexity of plasma chemistry.

To date, no relevant research has been reported on the TIG-based WAAW process for tin alloy deposition on the surface of inclined steel substrates. This study is therefore the first to examine the application of TIG-based additive manufacturing technology in the fabrication of steel/tin bimetallic structures. The effects of different process parameters on the morphology of deposited tin alloy layers, interfacial microstructure evolution and the mechanical properties of steel/tin bimetallic structures were systematically investigated under both horizontal and inclined substrate conditions. Furthermore, we identified the growth mechanism of the steel/tin interfacial microstructure, which provides a theoretical basis for the further expansion and application of TIG technology in the fabrication of dissimilar metals.

## 2. Materials and Methods

### 2.1. Materials

In this study, commercially available hot-rolled C45 steel was employed as the substrate material with customized dimensions of 200 mm × 150 mm × 10 mm. Featuring a fine-grained hot-rolled microstructure, C45 steel exhibits both exceptional structural rigidity and excellent weldability. A tin-based Babbitt alloy welding wire (grade: SnSb_11_Cu_6_) with a diameter of 1.6 mm was adopted as the deposition material. Tin serves as the matrix of this welding wire, which is strengthened by alloying elements such as antimony and copper. This design retains the excellent low-friction characteristics of tin while effectively enhancing the material’s high-temperature softening resistance. The detailed chemical compositions of the C45 steel substrate and tin alloy welding wire are listed in Table 1.

### 2.2. Principle of TIG-Based Wire Arc Additive Manufacturing

**Figure 1:**
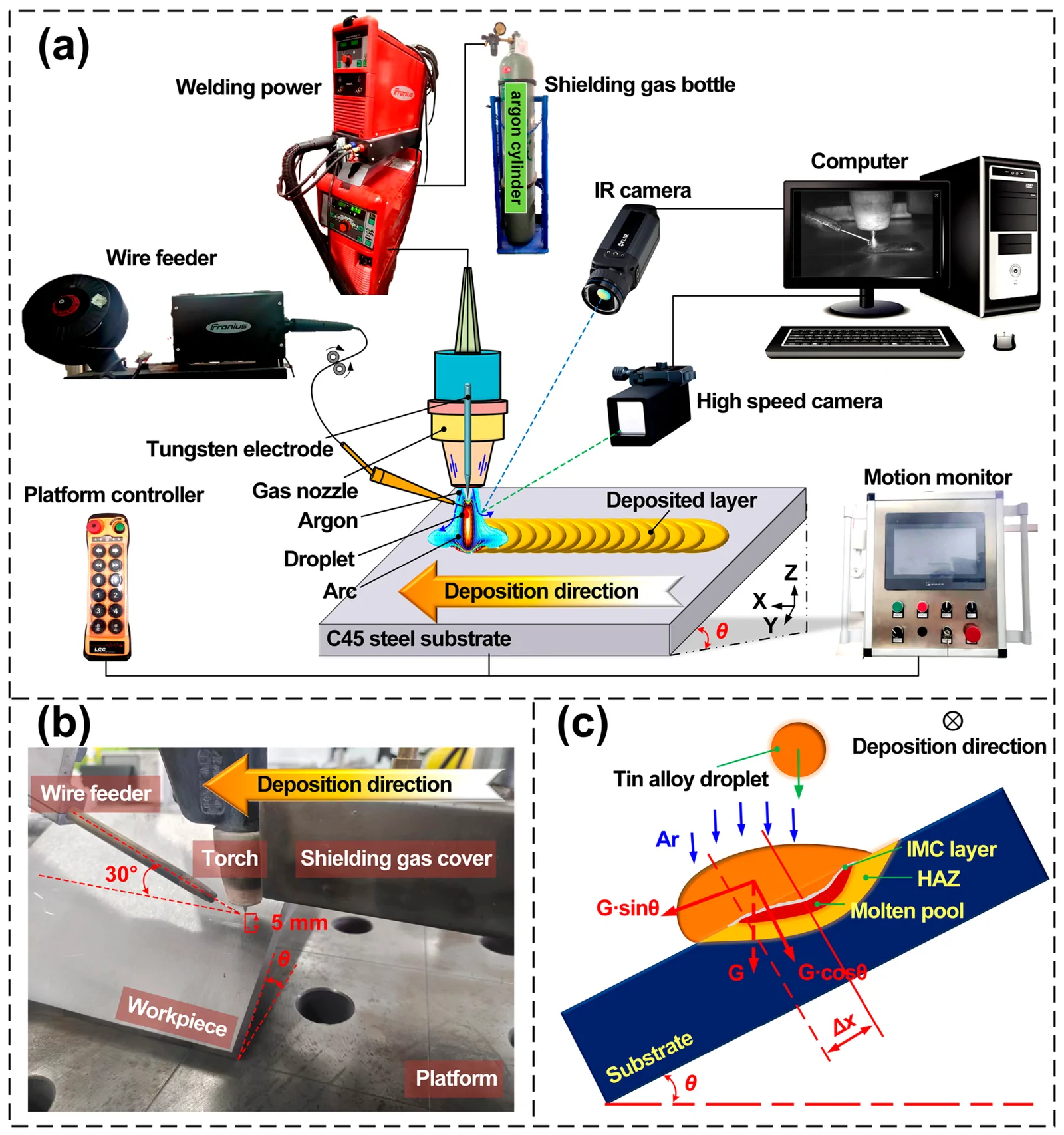
Schematic diagrams of the TIG-based wire-fed additive manufacturing system. (**a**) Schematic of the core TIG equipment configuration and deposition process; (**b**) schematic of the torch position and wire feeding angle; (**c**) force conditions of the molten droplet during impact on the substrate.

Figure 1a presents a schematic diagram of the TIG-based wire-fed additive manufacturing system, which mainly consists of a wire feeder, a welding power supply, a 3-axis CNC workbench, a molten pool monitoring camera, and an argon shielding system. The substrate subjected to oxide film removal treatment is fixed on the workbench, and the tin alloy welding wire is loaded into the wire feeder, followed by stable feeding into the arc zone via adjusting the rotational speed of the wire feed rollers. A direct current (DC) arc is ignited between the tungsten electrode and the substrate, and the arc heat melts the wire end to form molten droplets. The welding torch moves perpendicular to the substrate inclination direction, and the continuous solidification of the molten pool generates a single-track deposited layer. High-purity argon (99.99%) ejected from the nozzle forms a shielding gas curtain, preventing the oxidation of molten metal and deposited layers. A schematic cross-section of the as-fabricated specimen is displayed in Figure 1c. The combined effects of arc pressure, electromagnetic force, arc shear force, Marangoni force, droplet impact force, and gravity component due to the substrate inclination angle makes the melt flow within the molten pool more complex, leading to molten pool collapse and deposition instability. Under inclined substrate conditions, gravity G deflects relative to the central axis of the molten pool, decomposing into a G·sin*θ* component parallel to the substrate and a G·cos*θ* component perpendicular to it. This transformation converts the molten pool from a symmetric to a teardrop shape.

To estimate the effect of the substrate inclination angle, the three critical process parameters—welding current, arc voltage and wire feeding speed—were maintained at a constant level. During deposition experiments, the welding current and arc voltage used in the deposition experiments were set as 120 A and 13.5 V, respectively. The wire feed speed was 1.2 m/min, and the shielding gas flow rate was approximated as 15 L/min. The arc length of 5 mm was held constant by using arc voltage as the reference for a feedback control system, and the torch-to-workpiece distance was measured normal to the inclined substrate surface. The substrate remained fixed, while the TIG welding torch was superimposed in parallel using forward and backward movements. The wire feeder and the welding wire plate were mounted on the torch. The wire feeding angle relative to the local surface normal was set to 60° in each deposition experiment, the shielding gas geometry changed slightly with substrate inclination angle and the electrode axis remained fixed in the laboratory coordinate system. The chemical compositions and thermal properties of tin alloy and C45 steel are listed in Table 1, and the other process parameters are shown in Table 2. A total of 12 deposited specimens were prepared.

### 2.3. Characterization Analysis

Cross-sectional specimens with appropriate dimensions were sectioned from the stable forming zone perpendicular to the deposition direction via a BMT400C Wire Electrical Discharge Machining (EDM) device, which were utilized as metallographic characterization samples. The specimens were sequentially subjected to step-by-step water grinding using 150- to 3000-grit sandpapers until no obvious scratches appeared on the surface, followed by mechanical vibration polishing with diamond polishing pastes. Given the differential corrosion requirements of the steel/tin bimetallic interface, the C45 steel side was etched with a 4 vol% nitric acid alcohol solution, and the tin alloy side was subsequently corroded using a 10% ferric chloride hydrochloric acid solution (10 g FeCl_3_ + 20 mL HCl + 100 mL H_2_O).

The interfacial micromorphology of bimetallic structures, the thickness of IMC layers and the elemental distribution features of the tested samples under different inclination angles were characterized using a LEXT LS5100 Laser Scanning Confocal Microscope (LSCM) (Tokyo, Japan) and a SU5000 Field Emission Scanning Electron Microscope (FESEM) (Tokyo, Japan) equipped with an Energy Dispersive Spectrometer (EDS) (Tokyo, Japan). Image J (Version 1.54t) software was employed to measure the characteristic dimensions of the deposited layers. A Rigaku Smart Lab X-ray Diffractometer (XRD) (Tokyo, Japan) was applied to identify the phase composition of the interfacial micro-regions. Furthermore, Electron Backscatter Diffraction (EBSD) (Tokyo, Japan) technology was utilized to investigate the grain size, grain boundary orientation, and preferred growth direction near the bimetallic interface, with the testing parameters set as an accelerating voltage of 20 kV and a scanning step size of 0.47 µm. Microhardness measurements of the tin alloy and C45 steel at the bimetallic interface were conducted using a VIA-F Vickers Hardness Tester (Akita, Japan) under loads of 100 gf and 200 gf, respectively, with a dwell time of 10 s.

## 3. Results and Discussion

### 3.1. Macroscopic Forming Characteristics

**Figure 2:**
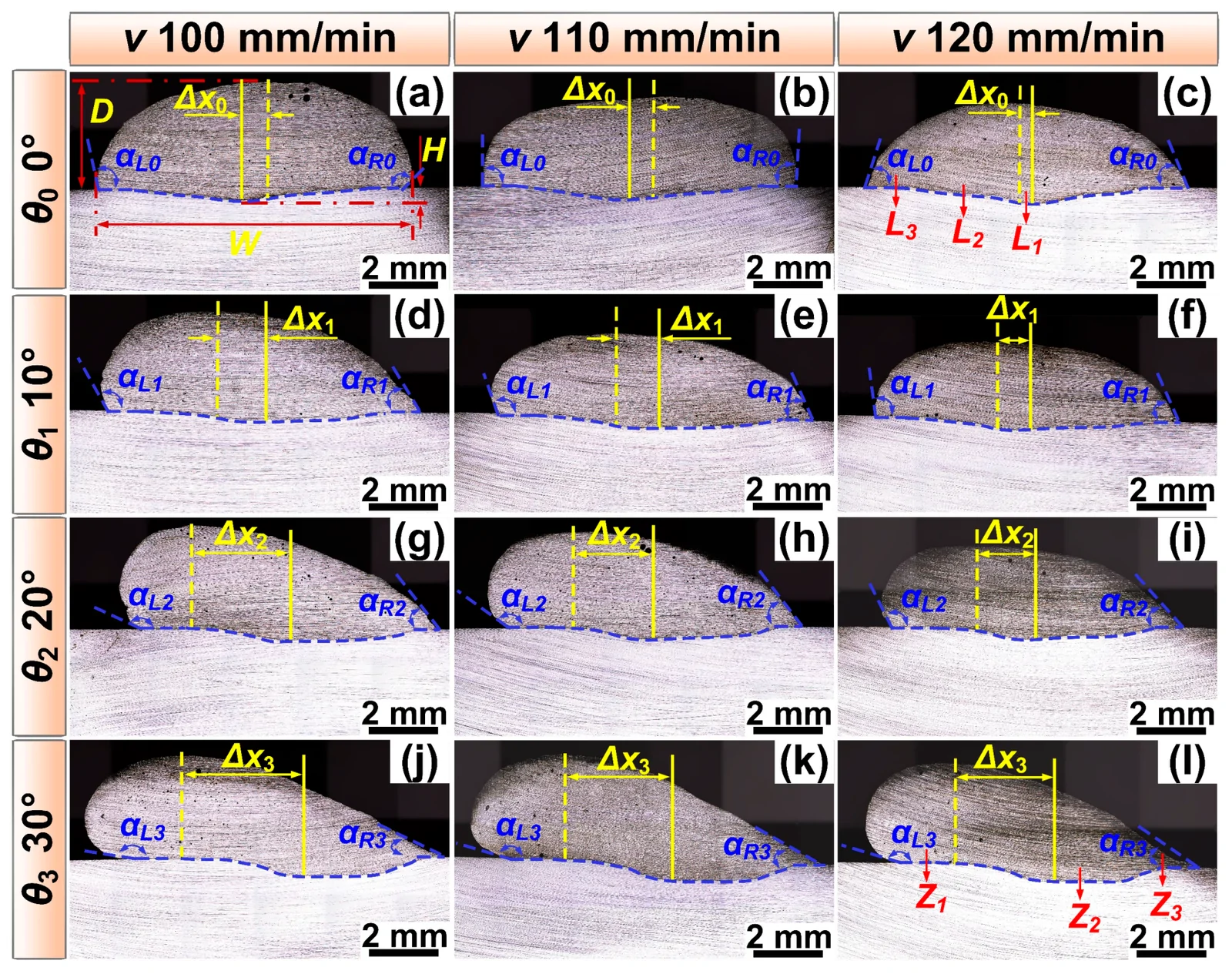
Cross-sectional morphology of single-track deposited tin alloy layers: (**a**–**c**) at a substrate inclination angle of 0° and deposition speeds of 100 mm/min, 110 mm/min, and 120 mm/min; (**d**–**f**) at a substrate inclination angle of 10° and deposition speeds of 100 mm/min, 110 mm/min, and 120 mm/min; (**g**–**i**) at a substrate inclination angle of 20° and deposition speeds of 100 mm/min, 110 mm/min, and 120 mm/min; and (**j**–**l**) at a substrate inclination angle of 30° and deposition speeds of 100 mm/min, 110 mm/min, and 120 mm/min.

The cross-sectional macroscopic morphologies of the prepared steel/tin bimetallic components under different process parameters are presented in Figure 2, showing the complete profiles of the deposited tin alloy layers; it can be seen that the interfaces between the C45 steel substrate and the deposited tin alloy layer do not occur on a straight line, but rather start in a concave-down manner and transition to a concave-up configuration where the TIG welding arc typically strikes the substrate surface. Macro-metallurgical defects near the bimetallic interfaces, such as porosity, inclusions and internal cracking, can be observed.

As shown in Figure 2c, only a small number of tiny micropores are distributed at the top of the deposited layer. Under a constant substrate inclination angle, increasing the deposition speed can significantly reduce the size and quantity of micropores at the top of the deposited layer. The increase in deposition speed does not just affect the deposition morphology; as the molten pool remains at a high temperature for a shortened period, this can speed up the pool’s flow, promote floating bubbles, and reduce the number of bubbles in the deposits. When the deposition speed remains unchanged, increasing the substrate inclination angle reduces the size of micropores but elevates their distribution density. Compared with the horizontal substrate, the micropore distribution shifts from the top center to the spreading rear edge (left side), whereas no obvious micropores are observed on the spreading front (right side). This phenomenon is mainly attributed to the insufficient arc energy supply on the left side, which leads to the tin alloy demonstrating a high melt viscosity and hinders gas escape, resulting in the formation of dense micropores. On the right side, the relatively small gap between the arc and the substrate leads to higher arc pressure, which can also promote the flow of the molten pool and increases the difficulty of bubble generation, thereby reducing the generation of pore defects.

### 3.2. Effects of Substrate Inclination Angle and Deposition Speed on Feature Dimensions of Deposits

To quantitatively analyze the influence of substrate inclination angle and deposition speed on the morphology of deposited tin alloy layers, several key characteristic parameters were introduced, including layer width (*W*), layer height (*H*), penetration depth (*D*), left and right contact angles (*α*_L_, *α*_R_) and cross-sectional offset (Δ*x*, defined as the relative offset between the highest point of the deposited layer and the central axis of the molten pool) [30]. The average offset rates and contact angle asymmetry coefficient *K* are defined by Equation (1) and Equation (2), respectively:

(1) $$s_{n}=\frac{\Delta x_{n}-\Delta x_{0}}{\Delta \theta_{n}-\Delta \theta_{0}},\;n=1,2,3$$

(2) $$K_{n}=\frac{\left|\alpha_{\mathrm{Ln}}-\alpha_{Rn}\right|}{\left(\alpha_{\mathrm{Ln}}+\alpha_{Rn}\right)/2}100\%,\;n=1,2,3$$

Here, *s* characterizes the offset rate of the deposited layer induced by the variation in substrate inclination angle, while *K* is used to evaluate the morphological asymmetry of the deposited layer; a larger *K* value indicates a more significant morphological asymmetry.

**Figure 3:**
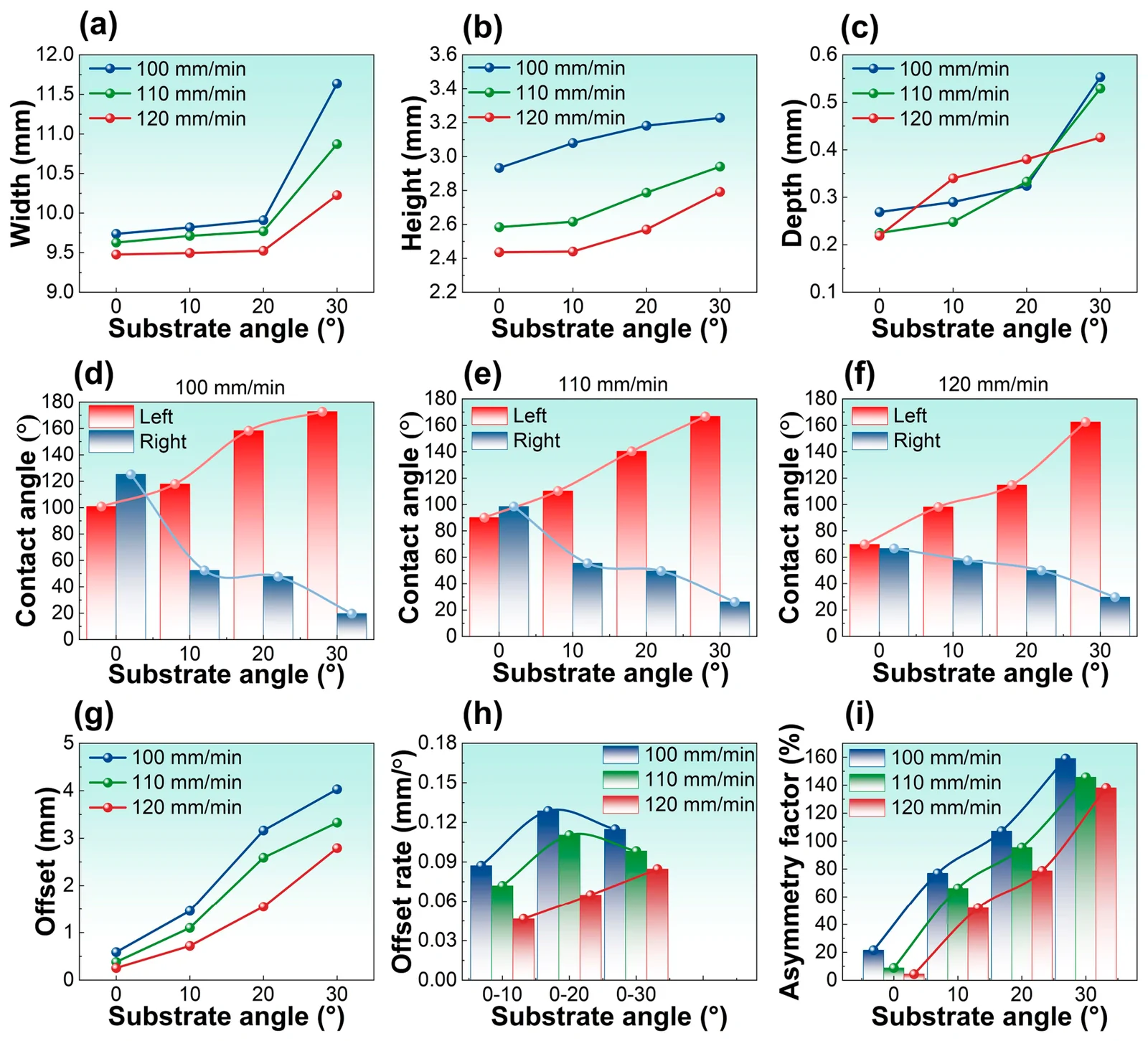
Variation curves of characteristic dimensions for single-track tin alloy deposited layers with changes in substrate inclination angle and deposition speed. (**a**) Layer width, (**b**) layer height, (**c**) penetration depth, (**d**–**f**) left–right contact angle of deposited layer, (**g**) offset of stacked layer, (**h**) average offset rate, (**i**) contact angle asymmetry coefficient.

The variation curves for the feature dimensions of the deposited layers with increases in the substrate inclination angle and deposition speed are shown in Figure 3. Here, the feature dimensions of deposited tin alloy layers and IMC thickness are counted in three replicates. Error bars were also provided to indicate the standard deviations in the two figures, but the length of the bars was too small compared to the scale of the variable mean, and hence all look identical.

It can be seen that at a fixed deposition speed, as the substrate inclination angle increases from 0° to 30°, *W*, *H*, *D*, *α*_L_, Δ*x* and *K* exhibit an overall upward trend, whereas *α*_R_ shows a continuous downward trend. Within the range of 0–20°, the gravitational component force exerts a weak effect on the molten pool morphology, resulting in a gentle increase in *W*. When the inclination angle exceeds 20°, the gravitational component force dominates the melt flow, aggravating the convergence of tin alloy melt toward the spreading rear edge and thus significantly elevating *W* and *H*. The increase in substrate inclination angle enlarges the difference between the left and right contact angles, directly driving the incrementation of *K*. Meanwhile, the spreading front approaches the arc center, concentrating the heat input and electromagnetic force to further promote an increase in penetration depth *D*. At a deposition speed of 120 mm/min, the molten pool solidifies rapidly, while at 100 mm/min and 110 mm/min, the excessive forward movement of the melt at an inclination of 30° leads to larger heat dissipation area and accelerated solidification at the rear spreading side, making *s* exhibit a trend of first increasing and then slowing down. At a fixed substrate inclination angle, the increase in deposition speed reduces heat accumulation, lowering the substrate temperature, accelerating molten pool solidification and weakening melt fluidity. This not only restricts the melt spreading range but also suppresses the gravity-driven asymmetric flow, ultimately resulting in simultaneous reductions in *W*, *H*, Δ*x*, *s* and *K*.

### 3.3. Interfacial Microstructure of Steel/Tin Bimetallic Structure

#### 3.3.1. XRD Phase Analysis

**Figure 4:**
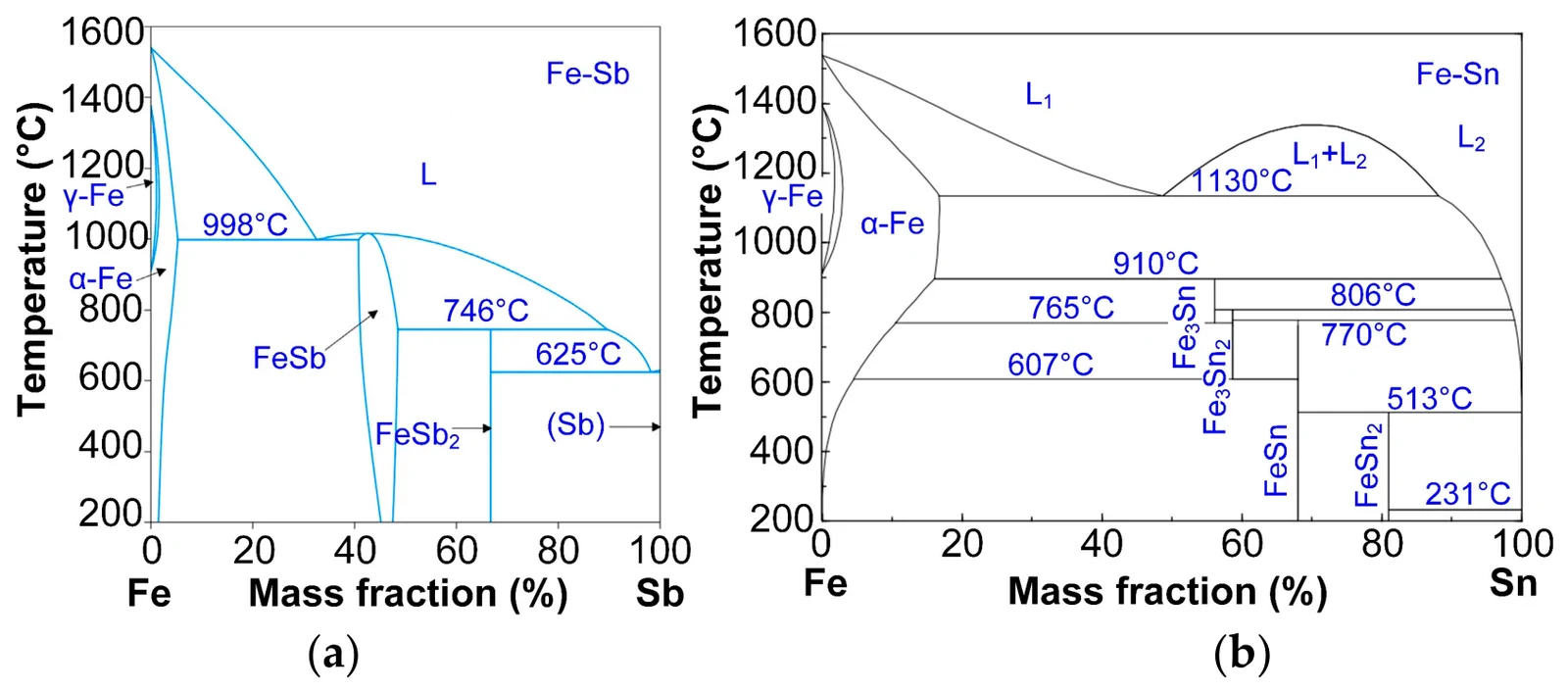
Phase diagram of binary alloys: (**a**) Fe-Sb, (**b**) Fe-Sn.

The Fe-Sb and Fe-Sn binary phase diagrams are presented in Figure 4. XRD line scanning tests were performed along the direction perpendicular to the steel/tin interface for specimens fabricated under both horizontal and 30° inclined substrates, and a deposition speed of 120 mm/min, so as to reveal the phase distribution laws in different characteristic regions.

**Figure 5:**
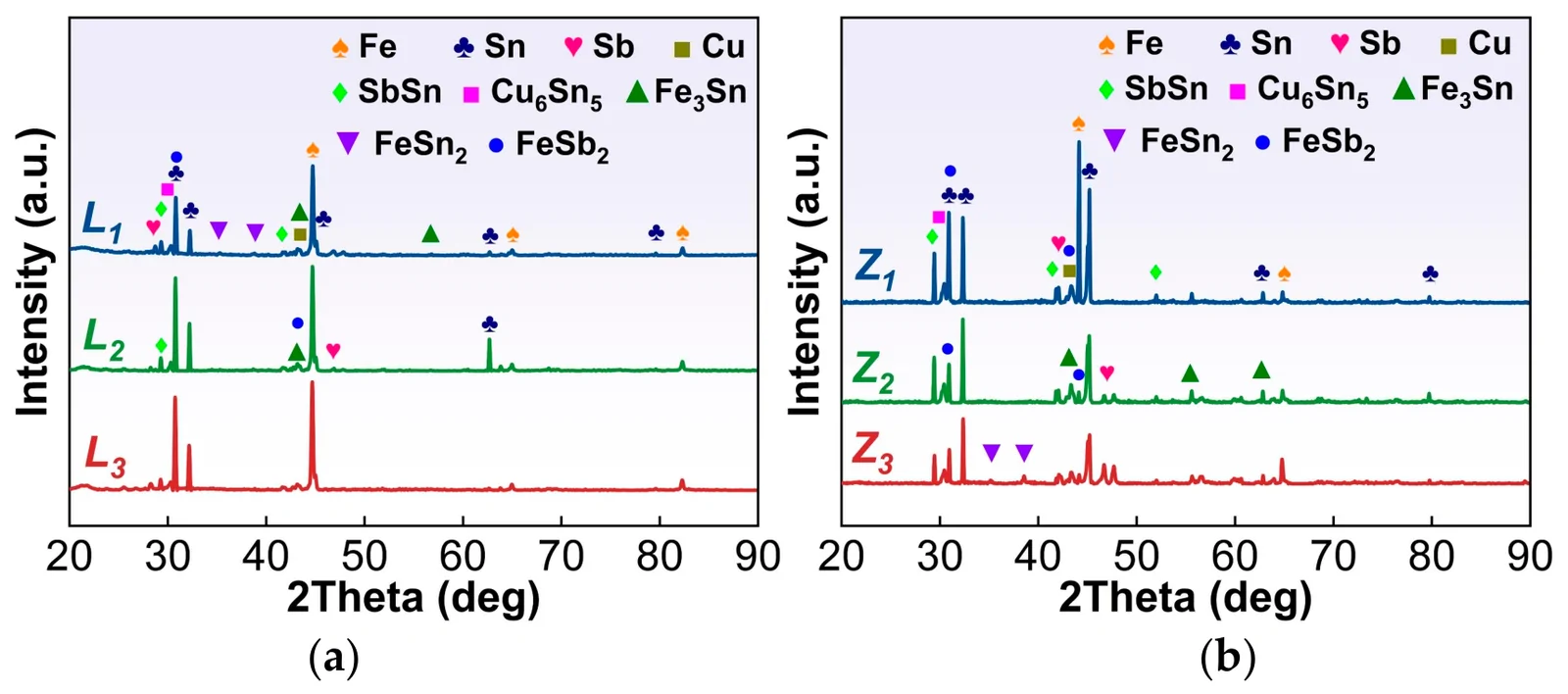
XRD patterns of different regions of the interface. (**a**) Horizontal substrate, (**b**) substrate inclination angle of 30°.

Figure 5 indicates that the phase constituents at the bimetallic interface mainly include the matrix phases Fe, Sn, Sb and Cu, as well as the intermetallic compounds Cu_6_Sn_5_, SnSb, Fe_3_Sn, FeSn_2_ and FeSb_2_. The results of the horizontal substrate specimen are shown in Figure 5a, where scanning lines *L*_1_, *L*_2_ and *L*_3_ correspond to the fusion zone, transition zone and left edge in Figure 2c, respectively.

It can clearly be seen that in regions *L*_2_ and *L*_3_, the relatively low temperature slows down the elemental diffusion rate, resulting in lower compound formation; meanwhile, the FeSn_2_ phase is not detected in these two regions. The XRD scanning results of the inclined substrate specimen are presented in Figure 5b, where *Z*_1_, *Z*_2_ and *Z*_3_ correspond to the spreading rear edge, fusion zone and spreading front in Figure 2l, respectively. *Z*_1_ is dominated by gravitational component force, with the maximum height of the deposited layer, and its phase diffraction peak intensity is significantly higher than that of other regions. *Z*_2_ is located in the high-temperature core zone of the arc, satisfying the formation conditions of the high-temperature Fe_3_Sn phase; this phase features excellent high-temperature stability and lower brittleness than FeSn_2_, which can effectively strengthen the interfacial bonding strength of the bimetallic structure [31,32]. *Z*_3_ is situated at the spreading front, and the rapid temperature drop is more consistent with the formation kinetic conditions of FeSn_2_.

#### 3.3.2. Effect of Substrate Inclination Angle on the Microstructure of the Bimetallic Interface

**Figure 6:**
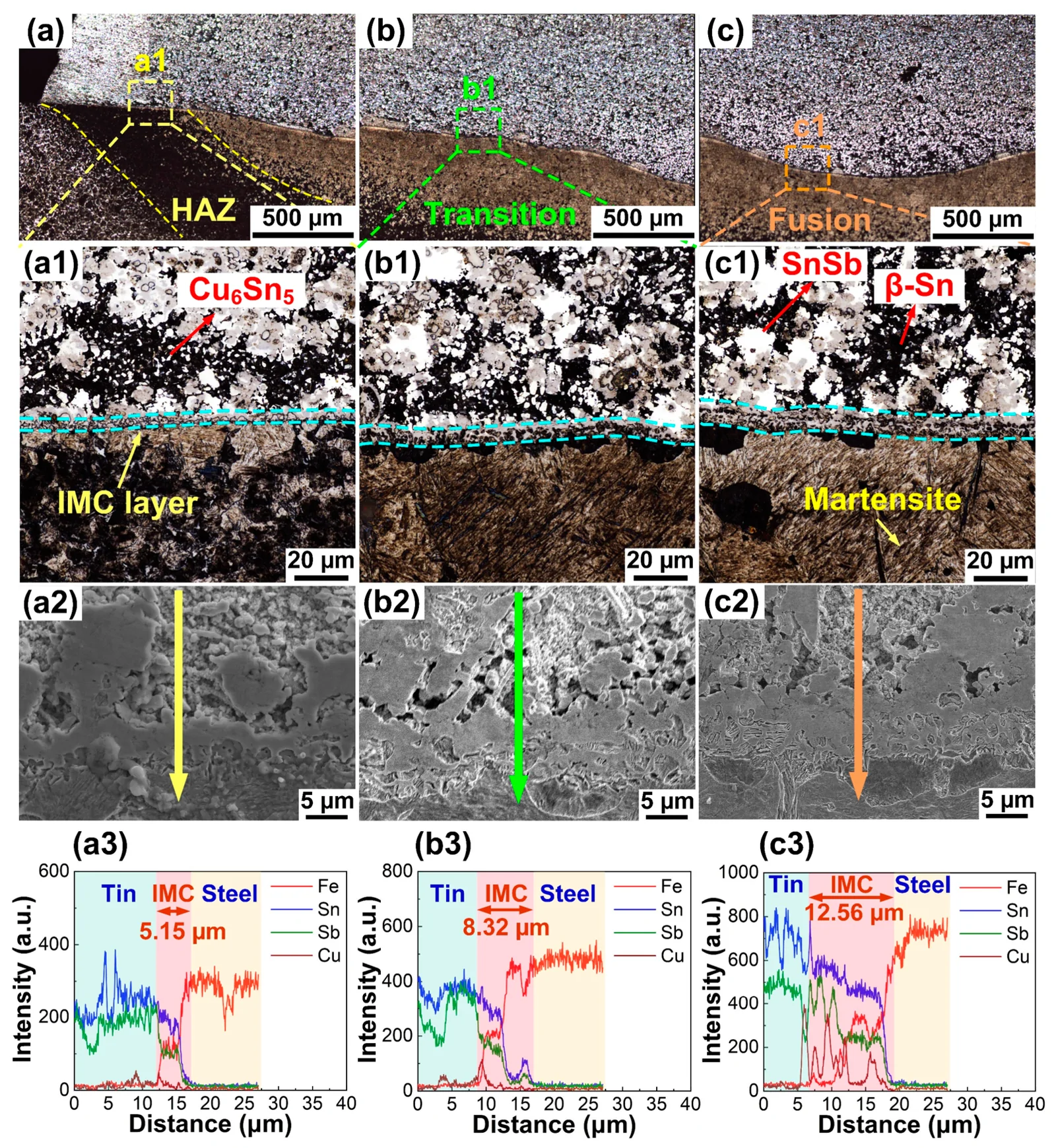
LSCM, SEM and EDS analyses of the heat-affected, transition and fusion zones in the horizontal substrate forming specimen interface. (**a**,**b**,**c**) Low-magnification LSCM microstructure, (**a1**,**b1**,**c1**) high-magnification LSCM microstructure, (**a2**,**b2**,**c2**) SEM morphology of the interface, (**a3**,**b3**,**c3**) EDS element distribution along the interface from the tin alloy to the steel side.

Figure 6 illustrates the microstructural morphologies of characteristic regions at the steel/tin bimetallic interface for the deposited specimen under horizontal substrate conditions. The microstructural evolution and solidification in the bimetallic interface and near-interface micro-regions are synergistically governed by the thermal history and the duration of heat accumulation. Metallographic results reveal that irregular white SnSb strengthening phases are dispersedly distributed on the tin alloy side, accompanied by fine ellipsoid-like Cu_6_Sn_5_ particles uniformly dispersed in the β-Sn matrix [33].

As depicted in Figure 6(a1), the microstructural characteristics of the heat-affected zone (HAZ) near the interface on the C45 steel side, which consists of dark flocculent troostite, fine acicular martensite and a small amount of ferrite. Constrained by the cooling rate, most austenite fails to reach the martensitic transformation temperature and preferentially undergoes diffusive transformation, forming troostite (a pearlite subtype) with fine interlamellar spacing; the retained austenite then transforms into fine acicular martensite during low-temperature cooling. In the fusion zone and transition zone shown in Figure 6(b1,c1), the near-interface microstructure is dominated by coarse high-carbon acicular martensite, indicating sufficient austenitization of the substrate surface layer. The interfacial region features high temperature, slow heat dissipation and a long high-temperature residence time. In addition to the IMC layer formed via elemental diffusion, a layer of fine black massive troostite is attached beneath it. Figure 6(a3–c3) display the distribution curves for the relative content of the main alloying elements near the interface in each respective characteristic region. It can be seen that the IMC layer thicknesses at the HAZ, transition zone and fusion zone are 5.15 µm, 8.32 µm and 12.56 µm in sequence, showing a gradual increasing trend from the spreading edge to the fusion zone. This phenomenon is correlated with the heat flux density distribution affected by the arc profile.

**Figure 7:**
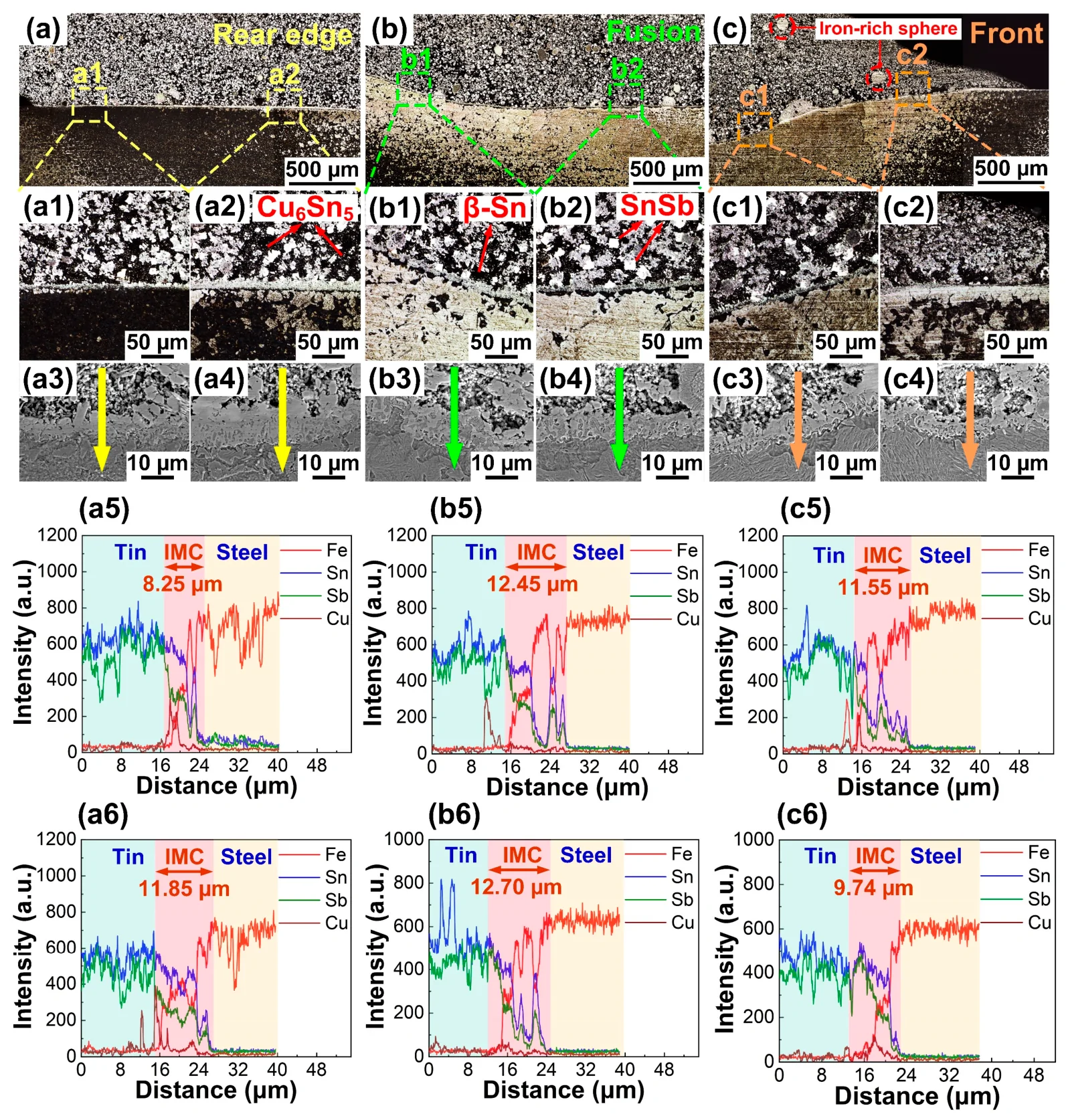
LSCM, SEM and EDS analyses of the spreading rear edge, fusion zone and spreading front of the 30° substrate inclination specimen’s interface. (**a**,**b**,**c**) Low-magnification LSCM microstructure, (**a1**,**a2**,**b1**,**b2**,**c1**,**c2**) high-magnification LSCM microstructure, (**a3**,**a4**,**b3**,**b4**,**c3**,**c4**) SEM morphology of the interface, (**a5**,**a6**,**b5**,**b6**,**c5**,**c6**) EDS elemental distribution along the interface from the tin alloy to the steel side.

Figure 7 presents the interfacial micromorphologies of characteristic regions in the deposited specimen under a 30° inclined substrate condition, where the gravitational component force exerts the most significant influence on the molten pool flow and thermal field.

#### 3.3.3. Evolution Patterns of the IMC Layer

**Figure 8:**
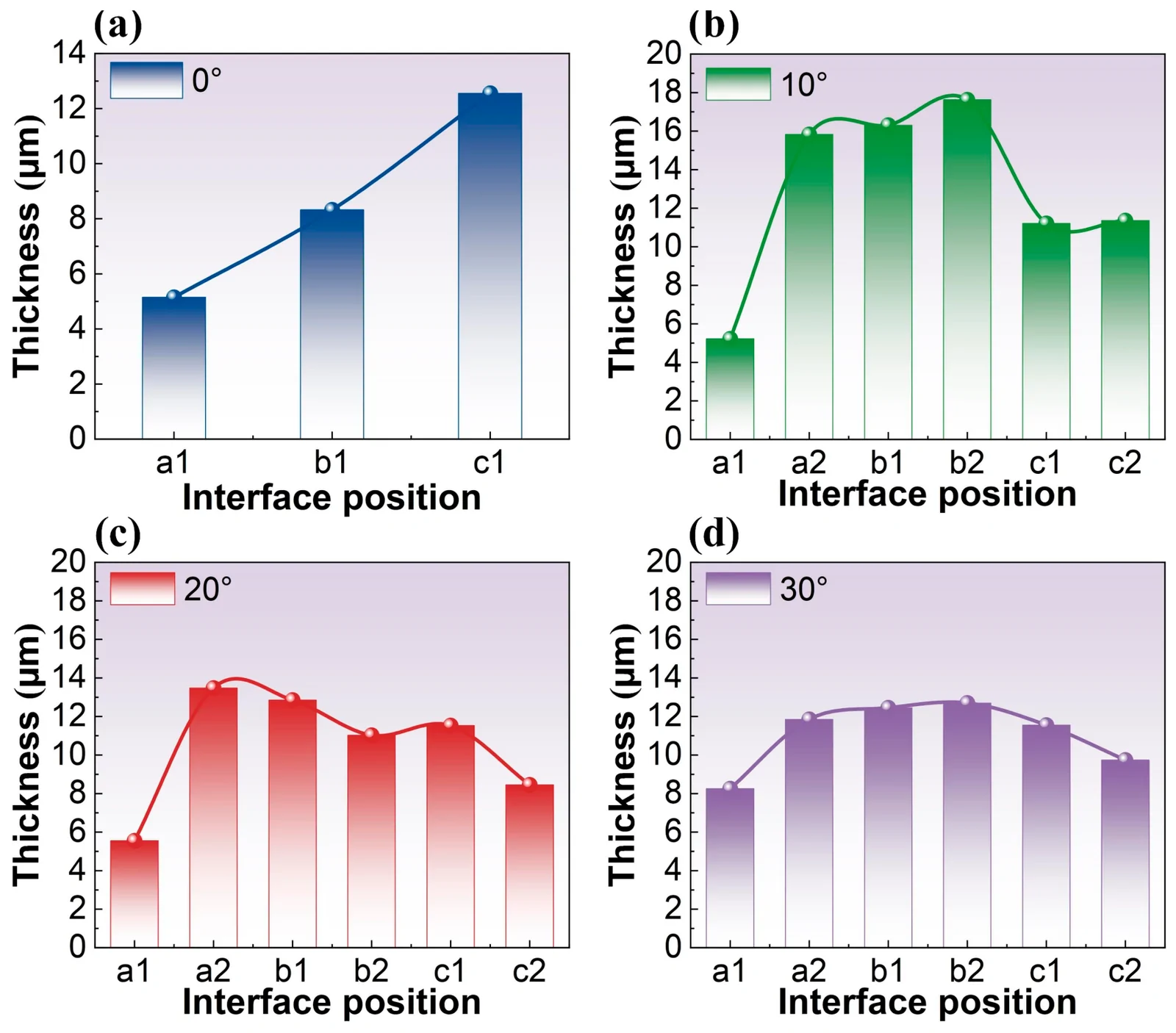
IMC layer thickness distribution patterns at different regions of the formed specimen’s interface under various substrate inclination angles: (**a**) 0° inclination angle, (**b**) 10° inclination angle, (**c**) 20° inclination angle, (**d**) 30° inclination angle.

Figure 8 presents the distribution of the IMC layer thickness in the characteristic regions of the formed specimen interface under different substrate inclination angles. Overall, it exhibits an evolutionary characteristic: from symmetric increase under the horizontal substrate, to central peak concentration under low inclination angles, and then to uniform distribution under large inclination angles. This development is related to the coupled regulatory effect of the substrate inclination angle on the TIG arc shape, heat flux distribution, and molten pool behavior.

The horizontal substrate condition is shown in Figure 8a. The arc presents a Gaussian symmetric shape, and the arc energy gradually decreases from the fusion zone to both sides of the edge, corresponding to an increasing trend of the IMC layer thickness from 5.15 µm at the left edge to 12.56 µm at the fusion zone. The low inclination angle of 10° is shown in Figure 8b. Here, a weak gravitational component force is applied to the molten pool, which breaks the symmetric distribution of the arc thermal field but does not induce violent flow. At this time, the arc shape does not undergo obvious deformation, and the fusion zone becomes the region with the most sufficient heat accumulation, leading to increased diffusion duration and rate for Fe and Sn atoms, with the peak IMC layer thickness reaching 17.63 µm. Although the heat input in the *L*-2 region is relatively low, the increased arc action area and duration also extend the diffusion time for Fe and Sn atoms, resulting in an IMC layer thickness of 15.82 µm. The 20° inclination angle is shown in Figure 8c; here, the gravitational component force is further strengthened, driving the molten pool to flow directionally toward the spreading rear edge, which leads to the dispersion of heat accumulation in the fusion zone. The peak IMC layer thickness decreases to 13.48 µm, and the difference in thickness between the front and spreading rear edge is significantly reduced. Finally, the limit inclination angle of 30° is shown in Figure 8d, where the gravitational component force reaches its peak, dominating the formation of a stable recirculation zone in the molten pool and promoting the uniform distribution of the arc heat flux. The overall difference in the IMC layer thickness distribution is further reduced, and the overall layer thickness is slightly higher than that under the horizontal substrate. This may be attributed to the increased inclination angle, which further expands the arc action area at the spreading rear edge and extends the element diffusion time, resulting in an IMC layer thickness of 8.25 µm at *L*-1, which is higher than that at the spreading rear edge under other inclination angles. Meanwhile, the high temperature of the fusion zone disperses to both sides, reducing the difference in IMC layer thickness and making the overall distribution exhibit an approximately normal distribution characteristic.

#### 3.3.4. EBSD Analysis

**Figure 9:**
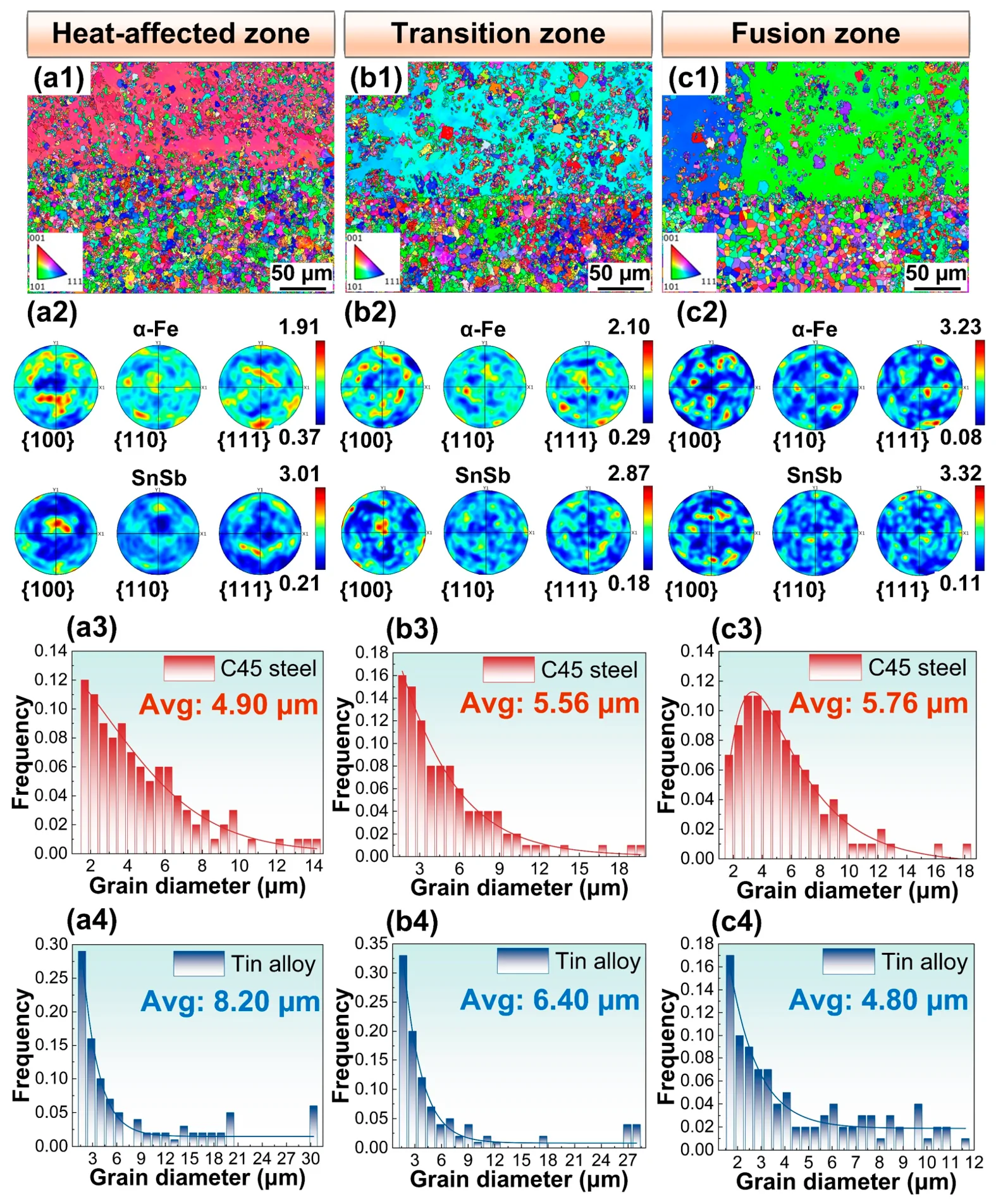
Grain size analysis of characteristic interfacial regions under horizontal substrate condition. (**a1**–**c1**) Inverse pole figures (IPFs), (**a2**–**c2**) {100}, {110} and {111} pole figures (PF) of α-Fe and SnSb, (**a3**–**c3**) substrate grain size distribution diagrams, (**a4**–**c4**) tin alloy grain size distribution diagrams.

Figure 9(a1–c1) show the inverse pole figures (IPFs) of the steel/tin bimetallic interface under the horizontal substrate condition. The α-Fe matrix grains on the steel side are randomly oriented, while the β-Sn matrix on the tin alloy side exhibits a significant preferred orientation.

The pole figures (PFs) of the matrix α-Fe phase and the SnSb phase are presented in Figure 9(a2–c2). When transitioning from the HAZ to the fusion zone, the maximum pole density of the two phases shows an increasing trend, suggesting a continuous enhancement of the preferred grain orientation. Here, the {100} crystal plane texture of the two phases in the HAZ exhibits a concentrated distribution, while the textures in the fusion zone are dispersed, with a significant improvement in texture uniformity. The grain size distributions on the steel side are presented in Figure 9(a3–c3). The average grain sizes of the corresponding regions are 4.90 μm, 5.56 μm and 5.76 μm, showing a gradual increase from the HAZ to the central area. This is ascribed to the high heat input and long high-temperature residence time in the transition zone and fusion zone, which realizes sufficient austenitization and provides thermodynamic conditions for grain growth. The grain size distributions on the tin alloy side are shown in Figure 9(a4–c4). The average grain sizes are 8.20 μm, 6.40 μm and 4.80 μm in sequence, which gradually decrease from the edge to the fusion zone of the deposited layer. The fusion zone possesses the maximum heat input and a rapid cooling rate, which further accelerates the nucleation rate. Meanwhile, the migrated Fe-rich microspheres are dispersedly distributed inside the deposited layer and further refine grains.

**Figure 10:**
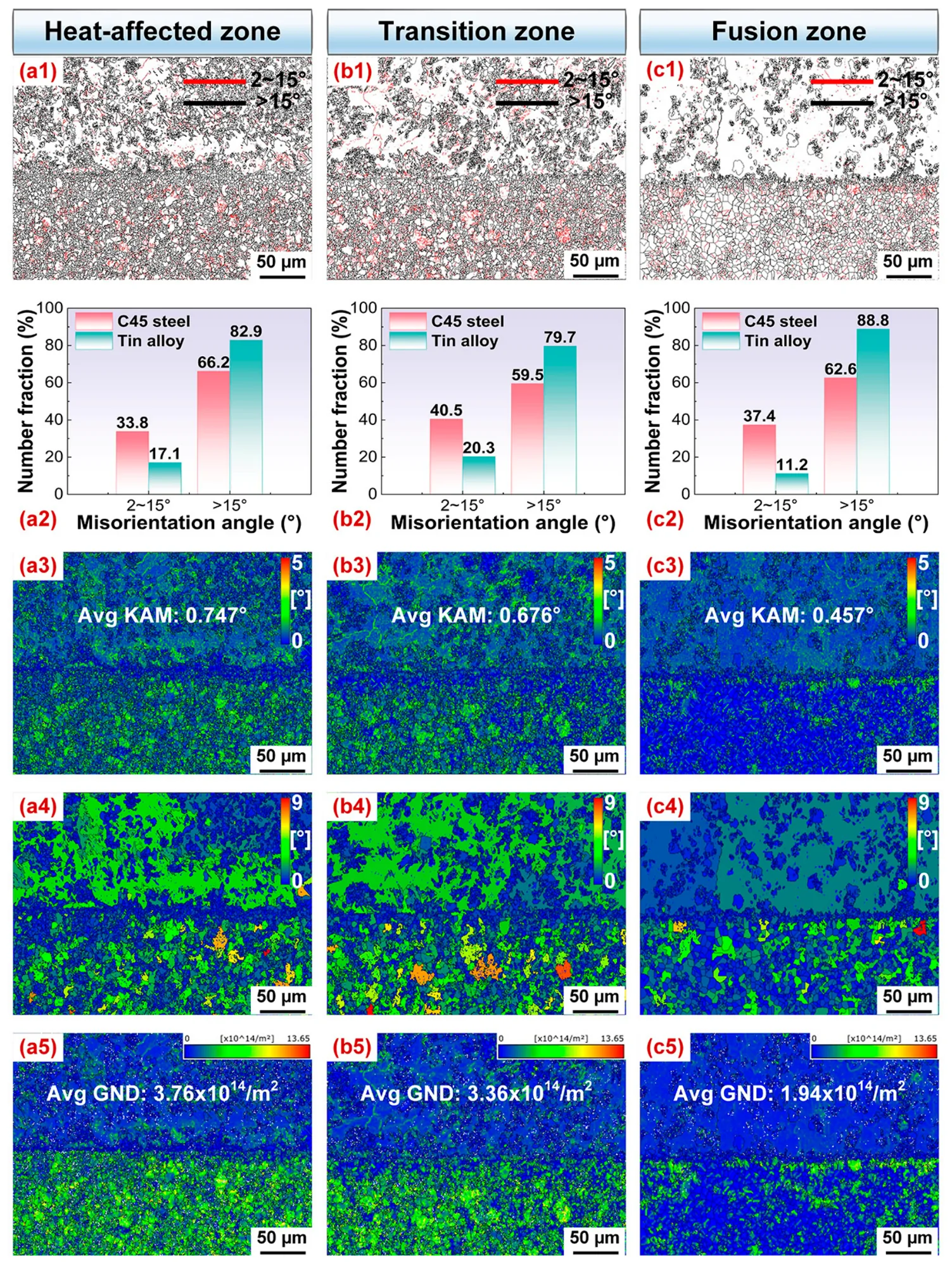
Grain orientation analysis of characteristic interfacial regions under horizontal substrate condition. (**a1**–**c1**) Interfacial grain boundary (GB) orientation difference, (**a2**–**c2**) statistical analysis of interfacial grain boundary angles, (**a3**–**c3**) interfacial kernel average misorientation (KAM), (**a4**–**c4**) interfacial grain orientation spread (GOS), (**a5**–**c5**) GND density maps for three feature regions.

Figure 10(a1–c1) show the grain boundary (GB) orientation difference distributions in the characteristic interfacial regions, where the red lines and black lines correspond to low-angle grain boundaries (LAGBs) and high-angle grain boundaries (HAGBs), respectively. The tin alloy side is dominated by HAGBs; under a high cooling rate, the nucleation rate surges and grains nucleate randomly, leading to a significant increase in orientation difference. A statistical analysis of the interfacial grain boundary angles is presented in Figure 10(a2–c2), with the percentage of HAGBs in the fusion region totaling 88.8%. A large number of HAGBs can effectively hinder dislocation movement and crack propagation, thereby significantly improving hardness and load-bearing capacity. In contrast, the steel side has a higher proportion of LAGBs (33.8~40.5%), which is conducive to enhancing matrix toughness. The kernel average misorientation (KAM) distributions are shown in Figure 10(a3–c3); KAM maps are used to characterize the dislocation enrichment degree and internal stress level inside grains and at their boundaries. The HAZ has the highest average KAM (0.747°), which is attributed to the large temperature gradient and high internal stress in this region, leading to hindered dislocation slip and massive accumulation. The fusion zone has the lowest average KAM (0.457°); due to concentrated heat input and a small temperature gradient, full austenitization followed by rapid solidification results in regular grain growth, sufficient stress relaxation at high temperature, and the lowest internal stress and dislocation density. The KAM values in the steel/tin interfacial region are generally low, indicating small internal stress and slight lattice distortion, which can inhibit the initiation and propagation of interfacial cracks and improve interfacial bonding strength. The grain orientation spread (GOS) distributions are shown in Figure 10(a4–c4); GOS maps are used to characterize the local strain degree of grains. The GOS of the tin alloy at the HAZ and transition zone is mainly blue and green, while the matrix also contains a small amount of yellow, indicating the higher deformation degree and stress concentration of the matrix grains. Figure 10(a5–c5) report the geometrically necessary dislocation (GND) density maps for the three feature regions of the as-deposited and tested samples. The GOS of the tin alloy in the fusion region is mainly blue, while the proportions of green and yellow in the matrix decrease, indicating that dislocations are fully relaxed during recrystallization and internal stress is effectively released. It is noteworthy that the misorientation was calculated between adjacent grains across the C45 steel/Babbitt interface.

**Figure 11:**
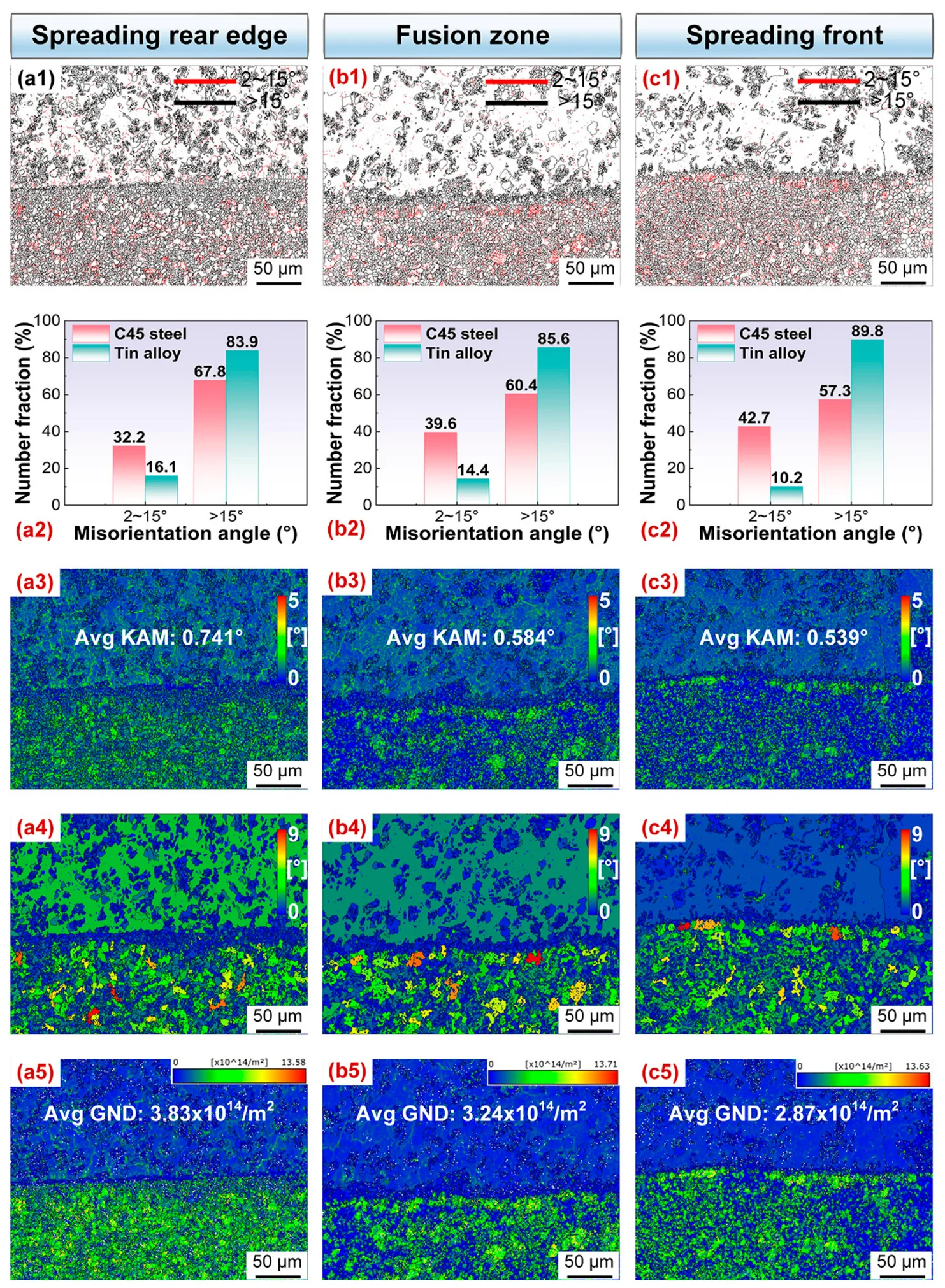
Grain orientation analysis of characteristic interfacial regions under 30° inclined substrate condition. (**a1**–**c1**) Interfacial grain boundary orientation difference, (**a2**–**c2**) statistical analysis of interfacial grain boundary angles, (**a3**–**c3**) interfacial kernel average misorientation, (**a4**–**c4**) interfacial grain orientation spread, (**a5**–**c5**) GND density maps.

Figure 11(a1–c1) illustrate the distributions of GB within characteristic interfacial regions at a substrate inclination of 30°. The tin alloy side predominantly comprises HAGBs; as shown in Figure 11(a2–c2), the percentage of HAGBs at the spreading front is as high as 89.8%. Such microstructural characteristics effectively hinder dislocation slip and grain deformation, which contributes positively to improving the load-bearing capacity of the bimetallic interface. Meanwhile, the fractions of HAGBs in the spreading rear edge and fusion zone of the substrate are increased correspondingly. The KAM distributions of the interfacial characteristic regions at the 30° inclined condition are presented in Figure 11(a3–c3), with the average KAM values determined to be 0.741°, 0.584°, and 0.539°, respectively. A decreasing tendency consistent with that obtained under the horizontal substrate is observed. The KAM value at the spreading rear edge is comparable to that of the HAZ for the horizontal sample, demonstrating severe lattice distortion, intensive dislocation aggregation, and insufficient internal stress relaxation in this region. Similar average KAM magnitudes are obtained for the fusion zone and spreading front. This phenomenon is attributed to the homogenized thermal field induced by the rightward arc deviation and molten pool recirculation, by which sufficient dislocation movement and stress relaxation are facilitated.

The GOS maps of the bimetallic interfacial characteristic regions under the 30° substrate inclination are shown in Figure 11(a4–c4). From the spreading rear edge to the spreading front, the GOS signals on the tin alloy side gradually transform from green (moderate strain) to blue (low strain). Nevertheless, no obvious variation in GOS contrast is identified in the corresponding substrate areas, and a small number of yellow regions associated with high strain are still retained. Compared with the horizontal substrate, lighter contrast and lower strain levels are detected in the fusion zone and spreading front at 30°. The inclined configuration enables a higher local heat input in these zones. Plastic strain is released via dislocation slip, and the degree of lattice distortion is reduced significantly. Similarly, Figure 11(a5–c5) display the GND density maps for the spreading rear edge, fusion zone, and spreading front. From the spreading rear edge to the front edge of the tin alloy, green regions with moderate strain gradually transition to blue regions with low strain, while no obvious change occurs in the substrate where local yellow regions with high strain still exist. Compared with the horizontal substrate, the fusion zone and spreading front present lighter color and lower strain degree. In general, the inclined substrate can reduce the internal stress at the bimetallic interface and effectively inhibit dislocation accumulation and crack propagation. Overall, the adoption of an inclined substrate enables internal residual stress to be reduced at the bimetallic interface and dislocation accumulation and crack propagation to be suppressed, guaranteeing the optimization of interfacial mechanical properties.

### 3.4. Microhardness Analysis

**Figure 12:**
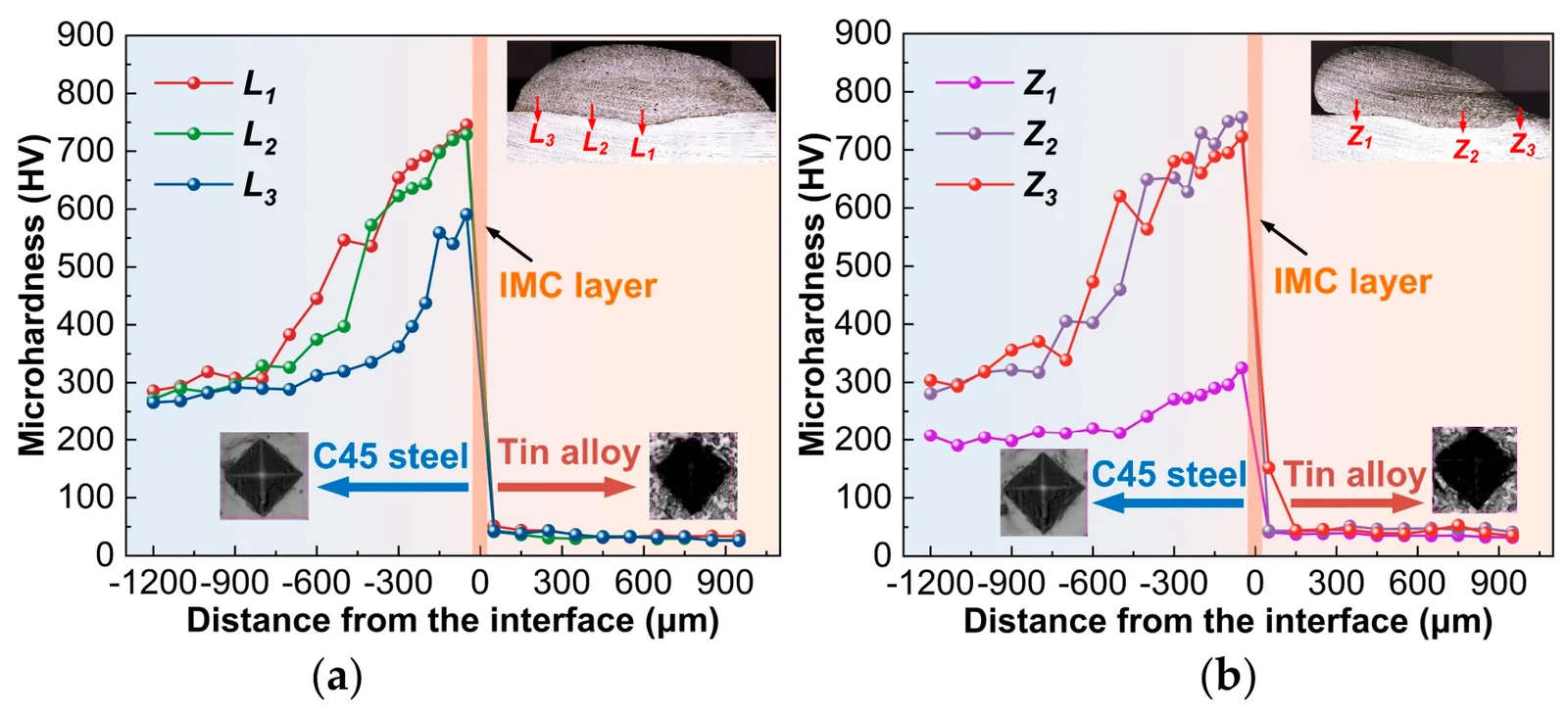
Microhardness distribution at the bonding interface of single-track single-layer steel/tin bimetallic components. (**a**) Specimen fabricated on a horizontal substrate, (**b**) specimen fabricated on an inclined substrate.

Figure 12 demonstrates the influence of substrate inclination angle on microhardness at three different interfacial regions. The reported hardness value for each test location is the average of at least three measurements. Figure 12a shows that, for the horizontal substrate, the Vickers microhardness measured near the interface on the steel side increases gradually from the steel matrix toward the bonding interface in regions *L*_1_ (fusion zone) and *L*_2_ (transition zone).

It can be seen that the hardness ranges are 286.1–745.6 HV and 271.6–729.2 HV, respectively, while a narrower hardness range of 265.8–590.9 HV is obtained in *L*_3_ (HAZ). The microhardness on the tin alloy side exhibits minor fluctuations and is maintained within the range of 26.0–51.2 HV. This behavior can be explained by the fact that regions *L*_1_ and *L*_2_ are subjected to intense arc heating, which promotes the generation of high-carbon acicular martensite and thick Fe-Sn IMC layers. Consequently, pronounced dislocation strengthening and second-phase strengthening are achieved. In comparison, the HAZ is primarily composed of troostite and fine acicular martensite; its reduced strengthening efficiency leads to a decline in peak microhardness. The inclined substrate condition is shown in Figure 12b; the peak microhardness values on the steel side in *Z*_2_ (fusion zone) and *Z*_3_ (spreading front) reach 755.8 HV and 722.8 HV, respectively, which are markedly higher than the peak value of 324.6 HV at *Z*_1_ (spreading rear edge). Similarly, low microhardness values are recorded on the tin alloy side. Regions *Z*_2_ and *Z*_3_ are situated adjacent to the arc center, giving rise to a large penetration depth and sufficient heat input. The formation of thick IMC layers and martensite-dominated microstructures in these zones is responsible for their higher peak hardness. Nevertheless, the spreading rear edge is located far from the arc with inadequate heat supply. Despite the occurrence of grain refinement, the microstructures consist mainly of troostite and ferrite, resulting in relatively low microhardness. Compared with the horizontal substrate sample, a marginally higher peak hardness is observed in the fusion zone of the inclined specimen, and a more significant hardness disparity exists between the spreading rear edge and the fusion zone. These variations are governed by the modification of arc heat flux distribution induced by substrate inclination. The average microhardness of the tin alloy side reaches 40 HV, which is greater than the 30 HV measured for the horizontal substrate. A maximum microhardness of 151.8 HV is identified near the interface. This enhancement is attributed to the higher volume fraction of Fe microspheres in deposited tin alloy. Just like the particle-reinforced composite, the hardness variations are closely related to the volume fraction of the particles. The hardness of Fe microspheres is significantly higher than that of deposited tin alloy, and under inclined substrate conditions, the closer the deposited tin alloy is to the bimetallic interface, the higher the volume fraction of the Fe microspheres is. Consequently, the deposited alloy near the bimetallic interface is reinforced with non-uniformly distributed Fe microspheres. In addition, the high proportion of HAGBs contributes to grain boundary strengthening, which further improves the microhardness.

## 4. Conclusions

This study was the first to employ the GTAW technology to fabricate steel/tin bimetallic components on substrates with different inclinations. The effects of the substrate’s inclination on interfacial forming morphology, IMC growth, microstructure evolution, and grain characteristics was systematically investigated. Our main conclusions are as follows:

(1) The formed quality of the steel/tin bimetallic components is excellent, with a defect-free metallurgical bond at the interface. For the horizontal substrate samples, the IMC thickness shows a symmetrical distribution with a thicker center and thinner edges; for the inclined substrate samples, the IMC layer significantly thickens along the spreading rear edge and the overall distribution becomes asymmetric, due to the inclination causing a longer residence time for the molten pool at the rear surface and allowing for sufficient element diffusion. As the inclination angle increases, the temperature field becomes more homogeneous, and the thickness difference in interfacial reaction layers among different regions is gradually narrowed.

(2) With the increase in substrate inclination angle, the spreading width, offset and contact angle asymmetry coefficient of the deposited layer rise simultaneously, and the deposited layer forms an asymmetric teardrop profile. Appropriately increasing the deposition speed reduces heat accumulation and shortens molten pool residence time. This can effectively inhibit the excessive downward spreading of the molten pool and avoid the degradation in forming quality caused by molten droplet sliding.

(3) Under inclined substrate conditions, SnSb reinforcing phases in the fusion zone appear as intact square particles and distribute uniformly. The asymmetric flow of the molten pool facilitates thorough mixing of dissimilar metals. Driven by surface tension, Fe-rich microspheres are dispersed inside the deposited layer, and partial microspheres adhere to the bonding interface, which effectively improves the overall microhardness of the deposition layer.

(4) At the substrate inclination angle of 30°, the spreading rear edge of the deposited layer deviates from the arc center, the heat flux density decreases and there is remarkable grain refinement. The fraction of HAGB at the spreading front reaches 89.8%, suggesting slight deformation of SnSb reinforcing phase grains. The KAM, GOS and GND in the fusion zone decrease, which is beneficial for reducing stress and strain.

Research findings will provide a better understanding of deposition process and bonding mechanism between deposited tin alloy and C45 steel, and can be used to give better control to some process parameters.

## Figures and Tables

**Table 1:** Chemical composition of the C45 steel substrate and tin alloy (wt%).

C45 steel	C	Mn	Si	P	S	Fe
	0.45	0.54	0.22	0.02	0.01	Bal.
Tin alloy	Sb	Cu	Pb	Fe	Bi	Sn
	10.75	5.89	0.01	0.01	0.01	Bal.

**Table 2:** Deposition process parameters.

Sample (No.)	Substrate Inclination Angle (°)	Deposition Speed (mm/min)
S1	0	100
S2	0	110
S3	0	120
S4	10	100
S5	10	110
S6	10	120
S7	20	100
S8	20	110
S9	20	120
S10	30	100
S11	30	110
S12	30	120

## Data Availability

The original contributions presented in this study are included in the article. Further inquiries can be directed to the corresponding authors.
